# Optimization of stimulus properties for SSVEP-based BMI system with a heads-up display to control in-vehicle features

**DOI:** 10.1371/journal.pone.0308506

**Published:** 2024-09-17

**Authors:** Hossein Hamidi Shishavan, Raheli Roy, Kia Golzari, Abhishek Singla, David Zalozhin, Danny Lohan, Muhamed Farooq, Ercan M. Dede, Insoo Kim

**Affiliations:** 1 Department of Medicine, University of Connecticut School of Medicine, Farmington, Connecticut, United States of America; 2 Department of Biomedical Engineering, University of Connecticut, Storrs, Connecticut, United States of America; 3 Toyota Research Institute of North America, Ann Arbor, Michigan, United States of America; NUST: National University of Sciences and Technology, PAKISTAN

## Abstract

Over the years, the driver-vehicle interface has been improved, but interacting with in-vehicle features can still increase distraction and affect road safety. This study aims to introduce brain-machine interface (BMI)- based solution to potentially enhance road safety. To achieve this goal, we evaluated visual stimuli properties (SPs) for a steady state visually evoked potentials (SSVEP)-based BMI system. We used a heads-up display (HUD) as the primary screen to present icons for controlling in-vehicle functions such as music, temperature, settings, and navigation. We investigated the effect of various SPs on SSVEP detection performance including the duty cycle and signal-to-noise ratio of visual stimuli, the size, color, and frequency of the icons, and array configuration and location. The experiments were conducted with 10 volunteers and the signals were analyzed using the canonical correlation analysis (CCA), filter bank CCA (FBCCA), and power spectral density analysis (PSDA). Our experimental results suggest that stimuli with a green color, a duty cycle of 50%, presented at a central location, with a size of 36 cm^2^ elicit a significantly stronger SSVEP response and enhanced SSVEP detection time. We also observed that lower SNR stimuli significantly affect SSVEP detection performance. There was no statistically significant difference observed in SSVEP response between the use of an LCD monitor and a HUD.

## I. Introduction

Recent advances in automobile technology have dramatically improved driver safety and in-vehicle convenience. However, it is important to acknowledge that controlling in-vehicle features through an onboard or head-down display and buttons while driving has the potential to distract the driver despite the value and benefits they provide. Approximately 25% of vehicle crashes in the last decade in the United States are estimated to result from the driver being inattentive or distracted [1]. In-vehicle activities, such as controlling the infotainment system, have recently created unsafe distractions for drivers, leading to an increase in the number of injuries and the severity of crashes [2,3]. Furthermore, the road safety risk is more pronounced for older adults, who take longer to complete in-vehicle tasks and experience increased visual distractions [4].

A brain-machine interface (BMI) has the potential to further enhance driving comfort and safety since it provides a direct path between the brain and external devices for alternative and augmentative communication. Several BMI paradigms have been developed in the past decades, and their applications have expanded to include in-vehicle controls and driver assistance [4–7]. Among the BMI paradigms, steady-state visual evoked potential (SSVEP)-based BMI has received increasing attention due to its noninvasiveness [8], high information transfer rate (ITR), zero calibration [9], and low BMI-illiterate rate [10]. Thus, SSVEP-based BMI applications have been widely used in BMI applications for vehicles, such as allowing the driver to select different driving modes (e.g., eco-mode for better fuel efficiency or a sport mode for higher performance) [11], mind-controlled driving that driver control certain aspects of the vehicle’s operation (e.g., steering, acceleration, or braking) to potentially allow for hands-free driving scenarios [12,13], and infotainment control such as changing the radio station [14].

To use SSVEP-based BMI systems in vehicle environments, it is crucial to achieve high accuracy and fast SSVEP detection time to minimize driver distraction when using the BMI systems while driving. In particular, visual stimuli properties (SPs) are critical factors in determining the performance of the BMI system [15,16]. Therefore, optimizing SPs can enhance the SSVEP signal quality (SNR) compared to the brain background activity, resulting in improved performance of the BMI system [17]. However, there is a lack of comprehensive understanding of the effect and interaction of SPs on SSVEPs, especially in the vehicle environment. For example, there are few studies on the effect of transparent glass in BMI research and whether modulating stimulus frequencies with background activity or ambient light could impact the strength of SSVEP response.

Further, the influence of other factors, such as monitor size and field of view (FOV), stimuli location and array configuration, and presentation platform (i.e., a HUD) on SSVEP response, has not been thoroughly investigated. Previously studied SPs also have contradicting results, such as the effect of stimuli color. For example, a study on stimuli color by Duszyk et al. [16] showed that dark colors (i.e., blue, green) evoke lower SSVEP responses in comparison to bright colors (i.e., white or yellow). However, another review study by Zhu [18] indicated that best-performing BMIs used green lights in their studies. In a recent study conducted by Zhang et al. [19], it was observed that the intensity of SSVEP response exhibited a gradual decrease as the light intensity increased. Their finding sheds light on the differences observed in experimental results and emphasizes the significance of stimuli in influencing SSVEP responses. Also, a finding by Ladouce et al. [20] suggests that reducing the amplitude depth (contrast) of low-frequency SSVEP stimuli can improve user comfort while maintaining high classification performance. Their finding is helpful, as in the past, using high-frequency stimuli was considered the only viable choice to improve visual comfort, but their classification performance was not competitive enough to design a reliable/responsive BMI. In line with this study, Xu et al. [21] evaluated the trade-off between decoding accuracy and subjective comfort level for various visual stimulus designs (contrast levels, frequency ranges, and temporal patterns) in a VEP-based BMI. The results showed that while low-frequency SSVEP stimuli had the highest decoding accuracy, they were rated as least comfortable. Conversely, high-frequency SSVEP stimuli were most comfortable but had the lowest accuracy. The study suggests code-modulated VEP (c-VEP) as a preferred stimulus for balancing decoding accuracy and comfort. For further investigation, an open dataset [22] recorded at stimulation frequencies ranging from 1 to 60 Hz provides a comprehensive resource for studying modulation depths and frequency ranges.

In addition, displaying visual stimuli on LCD/CRT screens, typically head-down displays, is not ideal in driving applications as it may impede the driver’s FOV or divert their attention away from the road, posing a potential hazard. In contrast, displaying visual stimuli on a head-up display (HUD) ensures that the driver’s FOV is not obstructed because the HUD reflects an image on the windshield, providing visual stimuli within the driver’s immediate FOV [23]. Thus, recent SSVEP-based BMI studies presented visual stimuli on HUD [24–28]. However, previous studies that used HUDs for SSVEP detection faced several challenges. For example, a prolonged stimulus time (longer than 12 seconds) was required to obtain higher accuracies [4]. In addition, some participants couldn’t drive the simulated vehicle because of poor SSVEP accuracy [5]. Neither of the studies compared the outcomes using a conventional benchmark in SPs.

To address those challenges, we believe that the impact of the SPs presented on a HUD on the strength of SSVEPs must be investigated because different platforms for displaying stimuli, such as a monitor or projector, bring varying combinations of light to sensory input, which can affect the information received by the visual system [17,29]. Furthermore, the SPs on the windshield can affect eye comfort and SSVEP response differently [16]. Nevertheless, further research is needed to elucidate the usability and safety of an SSVEP-based BMI system that uses a HUD. Therefore, in this study, we investigated various SPs projected on the transparent glass (windshield) and compared the performance of an SSVEP-based BMI system that uses a HUD for displaying visual stimuli in a driving simulation environment. Additionally, we conducted experiments with an LCD monitor to compare the results obtained from experiments with an HUD.

Since the stimuli’ characteristics and properties significantly affect SSVEP-BMI performance, and their implementation should be optimized for each BMI system environment [30], we designed the BMI system in a simulated vehicle environment with an actual vehicle windshield and seat. Then, we evaluated several SPs that could significantly influence the SSVEP response, including the duty cycle of visual stimuli, the location of the stimuli on the screen, array configuration and geometric size of icons, the color of icons, and signal-to-noise ratio (SNR) of visual stimuli.

In the broader scheme, the BMI-based system employs optimized SPs in a multi-layered approach. User goes through a primary menu to select in-vehicle features, then tune settings in corresponding sub-menus. For instance, to activate the navigation system, users select "Navigation" from the primary menu and then choose from pre-stored addresses or other navigation settings in the second layer. The number of layers can be justified by the variety of settings available.

Section II describes the details of SPs studied in this article. We recruited 10 human volunteers for the experiments and analyzed the EEG signals using power spectral density analysis (PSDA), canonical correlation analysis (CCA), and filter bank CCA (FBCCA), which are widely used to detect SSVEPs in BMI studies. We conducted different sets of experiments to systematically assess SSVEP responses for both ideal and non-ideal (i.e., closer to real-world) conditions. Sections III and IV explain the experimental design and analysis methods in great detail. Section V presents the results of the experiments, and Section VI discusses the findings of this study.

## II. Visual stimuli properties

In this study, a total of eight different SPs were investigated, with a reference SP selected as a baseline. The reference SP had a size of 20.25 cm^2^, a duty cycle of 50% with a square wave, B/W color, a 1×4 array configuration, and was located in the center. It is worth noting that these properties were selected based on our previous experiments and other reports [16,17,26,31,32]. The remaining SPs were changed one at a time to investigate their influence on the SSVEP. Initially, the reference SP with high and low SNR stimuli are studied using both the LCD monitor and HUD. Subsequently, four different stimuli frequencies, four different duty cycles, two different screen locations, two array configurations, seven different colors, and four different icon sizes are considered for HUD. Table 1 provides a summary of the SPs discussed in this study.

**Table 1:** Summary of the SPs investigated.

SPs	Details
**Display Type**	LCD Monitor, HUD
**Stimuli Frequency (Hz)**	6.67, 7.5, 8.57, 10
**Duty cycle (%)**	[14–25], [28–38], [45–57], [62–72]
Location Array Configuration	Center, Bottom 1×4, 2×2
**Color**	Blue, Red, Green, Magenta, Cyan, Yellow, B/W
Size (cm^2^)	5.06, 20.25, 36, 81
**Stimuli SNR**	30 dB, 70 dB

### A. Visual stimuli frequency

For quick and reliable detection of SSVEPs using a typical EEG headset and signal processing methods, stimuli frequencies need to be limited between 6 and 12 Hz [33]. This frequency range is further constrained by the picture frame rate (PFR) to evoke stronger SSVEPs at desired frequencies. Thus, this study selected stimuli frequencies of 6.67 Hz, 7.5 Hz, 8.57 Hz, and 10 Hz, which were accurately displayed with nine, eight, seven, and six frames when the PFR was set to 60 frames/second (fps) [34].

### B. Duty cycle

**Fig 1:**
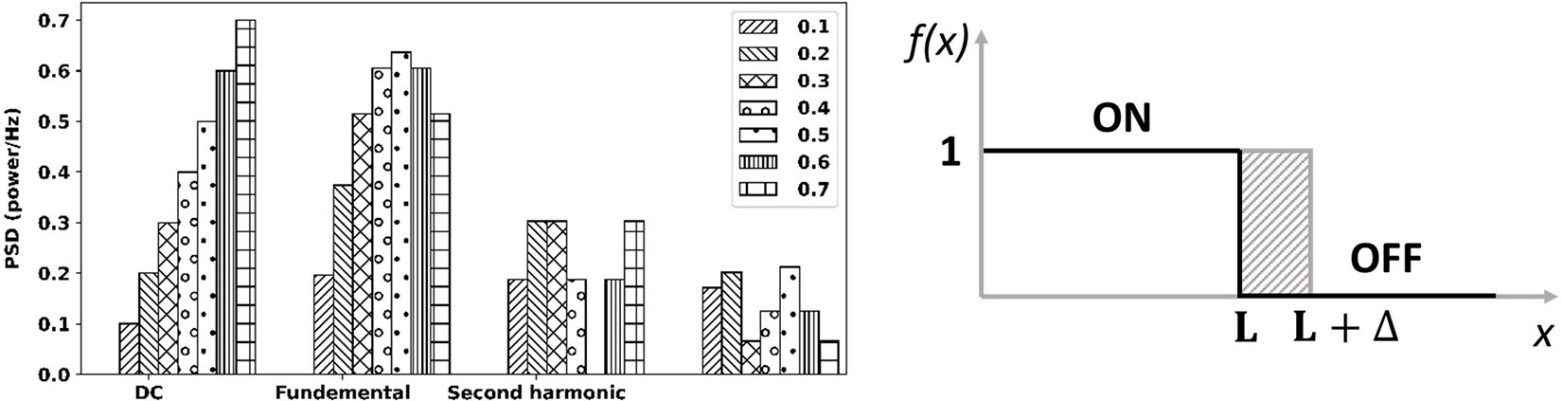
Calculated power spectrum of a square wave with duty cycles from 0.1 to 0.7 (left) and the proportion of ON and OFF states in one cycle/frame (right).

Adjusting the duty cycle in visual flicker changes the proportion of ON and OFF states in a frame-based realization. Due to the temporal integration of human vision (Bloch’s law: perceived contrast is linearly determined as a function of stimulus contrast and duration [31]), the duty cycle directly affects the strength of SSVEP by altering the amplitude of fundamental, harmonic, and DC values of stimuli frequencies [31]. Fig 1 shows the power spectrum for a square-wave function with different duty cycles calculated from Eq 1. The power spectrum amplitudes have noticeable differences depending on the duty cycle.


f(x)=a02+∑n=1∞[ancos(nπxL)+bnsin(nπxL)], (1)

a0=L+ΔL,an=(−1)nnπsin(nπxL),bn=1π(1+(−1)n−1cos(nπxL))

This equation demonstrates that a square wave with a 50% duty cycle (Δ = 0) would only have odd harmonics (i.e., 1st, 3rd, and 5th) in the power spectrum, while even harmonics would be absent. As detecting SSVEPs can be better accomplished in lower-order harmonic frequencies due to their high energy, a higher preference is given to these harmonics in BMI systems [5].

While higher duty cycles (shorter OFF state) can enhance visual comfort [8], this does not necessarily mean that they would also evoke a greater amplitude of SSVEP. Also, the influence of the duty cycle on SSVEP may vary for different target frequencies [9]. As frame-based control limits the accuracy of duty cycle setting, we employed duty cycle bins, where each bin represents a different range of frame-based duty cycle for stimuli frequencies. For instance, 6.67 Hz of stimuli require nine picture frames and cannot have a duty cycle of 50% without averaging several seconds of signal. Therefore, the closest ratio for the frames would be 56% (i.e., five frames on, four frames off), which we placed in the [45–57%] bin. Following the same approach, we created four bins to include all possible frame combinations, as summarized in Table 2.

**Table 2:** Duty cycle bins for target frequencies.

Frequency (Hz)	# of Frames	On/off frames (ratio in %)			
**6.67**	9	2/7 (22)	3/6 (33)	5/4 (56)	6/3 (67)
**7.5**	8	2/6 (25)	3/5 (38)	4/4 (50)	5/3 (63)
**8.57**	7	1/6 (14)	2/5 (29)	4/3 (57)	5/2 (71)
**10**	6	1/5 (17)	2/4 (33)	3/3 (50)	4/2 (67)
	Range	14–25%	28–38%	45–57%	62–72%

### C. Location, array configuration, and FOV

By directing the eye to different locations on the screen, the stimulus is projected to different locations on the retina relative to the fovea. Research has shown that these distinct patterns are sufficient to enable a classifier to identify which stimuli the user is attending to based solely on the location of the stimuli [35]. Therefore, we investigated the reported impact of retinotopic mapping on visual brain areas by examining how the center and bottom locations, using 1×4 and 2×2 array configurations, could affect SSVEP power distributions. The upper center and left sites were excluded due to concerns regarding driver distraction, direct sunlight, and the impact of protective sunshades on the windshields.

**Fig 2:**
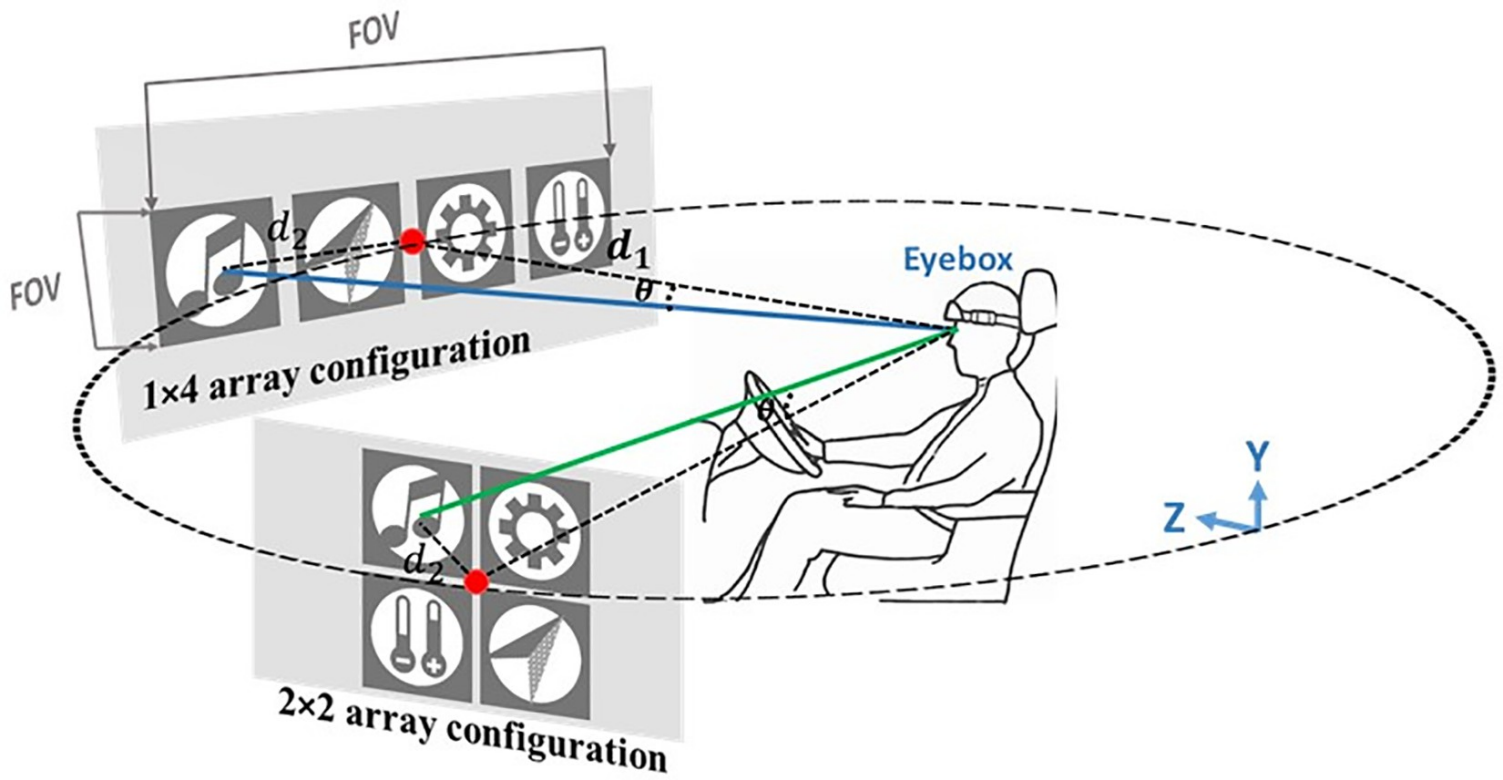
Presentation of stimuli on the HUD and subject’s eyebox.

The angle *θ*, from the fixation point (i.e., the point in front of the subject’s nose) to the center of the visual stimulus depends on the subject’s distance from the screen (*d*_1_) and the icon’s distance from the fixation point (*d*_2_), as shown in Fig 2. The viewing angle of the four icon stimuli is presented in Table 3. The distance between the subject and the display was 95 cm and 75 cm for the HUD and monitor, respectively. The FOV for the monitor was 8° × 1°, and for the HUD was 12° × 2°.

**Table 3:** The angle of the stimuli from the center and eyebox.

	1×4 Center LCD	1×4 Center HUD	1×4 Bottom HUD	2×2 bottom HUD	2×2 Center HUD
**Music**	3°	4°	7°	3°	1°
**Navigation**	1°	1°	3°	3°	1°
**Settings**	1°	1°	3°	3°	1°
**Temperature**	3°	4°	7°	3°	1°

### D. Color

High luminance, associated with higher contrast between color and background, can cause greater pupil constriction and reduce the amount of light entering the pupil that controls the amplitude of SSVEP [10]. Moreover, different types of photoreceptors in the retina have different spectral sensitivity, and contrast differences and color signals influence pupil response differently. This means monochromatic and combined monochromatic light have varying influences on the strength of the pupillary light response, depending on the wavelength. Furthermore, the Helmholtz-Kohlrausch (HK) effect can also modulate the pupillary response, where the different perceptions of the brightness of equiluminant stimuli affect the SSVEP response [10,11]. A better understanding of luminance perception and how different colors affect SSVEP response can help account for color contribution in stimuli design, improve accuracy, and reduce detection time. In this study, we examined three original colors (blue, red, and green), a combination of two original colors (magenta, cyan, and yellow), and black and white (B/W).

### E. Size

By increasing the stimuli size, more light enters the subjects’ FOV, which means that more information is detected by the visual sensory circuit [12]. This results in increased neuronal activity and a stronger evoked response. We studied the effect of stimulus size by using squares of different sizes projected on the windshield: 5.06 cm^2^ (1.35°, 77’), 20.25 cm^2^ (2.69°, 155’), 36 cm^2^ (3.6°, 206’), and 81 cm^2^ (5.38°, 310’). Note that values in parenthesis approximate vertical angle in degree and radian when viewed by the subjects from a fixed distance of 95 cm. We chose these sizes based on the receptive field (RF) size in the visual system [12].

### F. Visual stimuli SNR

**Fig 3:**
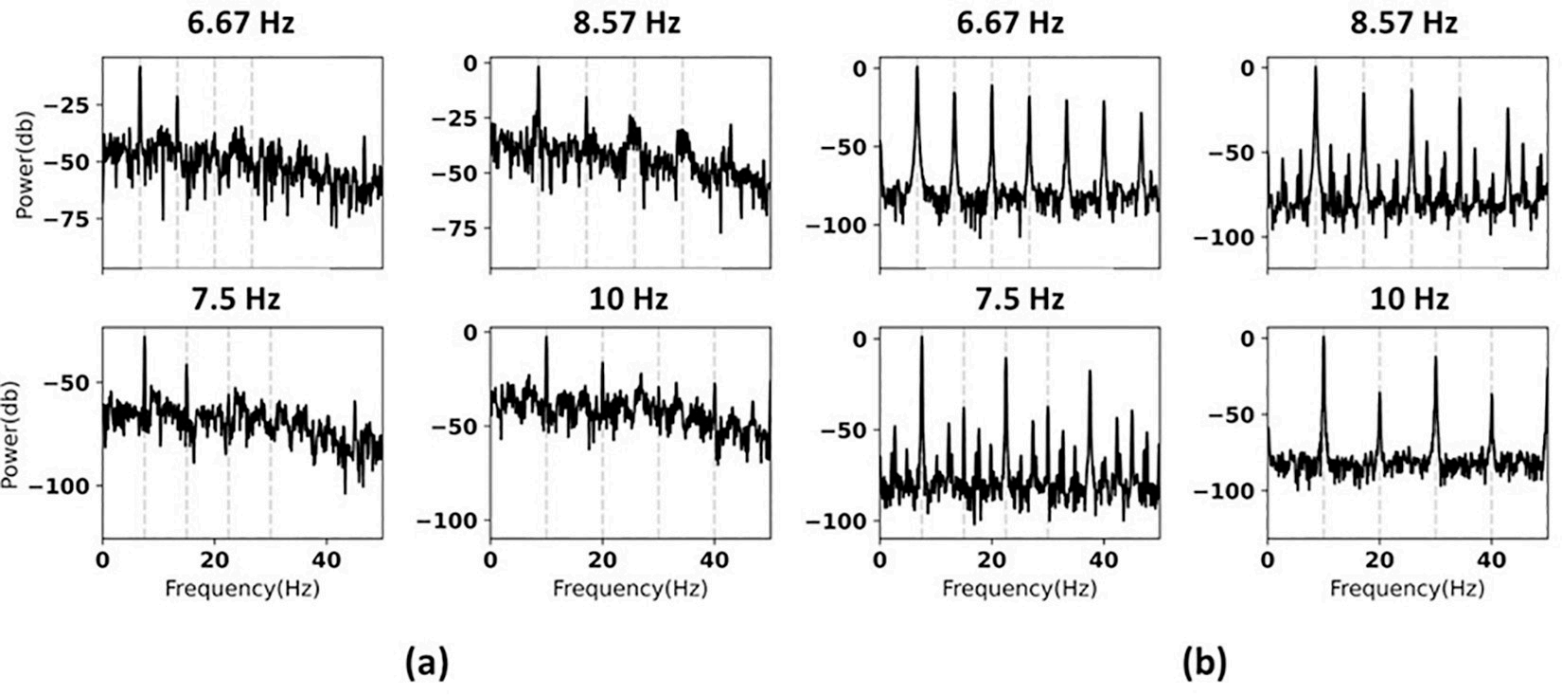
Power spectrum density of the visual stimuli for (a) low SNR and (b) high SNR.

When visual stimuli are projected onto the windshield, the driver’s attention may be drawn to the surrounding environment, causing background activity to modulate with the stimuli. This modulation can introduce unwanted frequency components in the driver’s field of vision, reducing the SNR of stimuli. Thus, in this study, we also investigated the effect of low and high SNR visual stimuli on SSVEP magnitude when using a windshield HUD. We measured the intensity of the stimuli light on the glass screen using a photodiode to assess the quality of the rendered stimuli frequencies. Fig 3 shows frequency spectra of the target frequencies in two different conditions, resembling stimuli in real-world and ideal conditions, respectively. In the first experiment, we used low SNR stimuli, which could be attributed to imprecise frequency rendering, unwanted environmental distractors (e.g., dirty windshield), and inaccurate PFR control. In contrast, we used high SNR stimuli in the second experiment, with the signal of interest around 70 dB above the noise level (Table 4). In this study, SNR is defined as $10{log}_{10}\frac{P_{signal}}{P_{noise}}$, where *P*_*signal*_ and *P*_*noise*_ denote the power of the signal and noise at the target frequency and its harmonic, respectively.

**Table 4:** SNRs of the visual stimuli.

Frequency (Hz)	Low-SNR Stimuli (dB)		High-SNR Stimuli (dB)	
	Fundamental	2^nd^ Harmonic	Fundamental	2^nd^ Harmonic
**6.67**	32	15	70	60
**7.5**	31	12	75	10
**8.57**	31	12	79	60
**10**	33	16	70	45

## III. Experimental method

### A. Windshield and HUD

**Fig 4:**
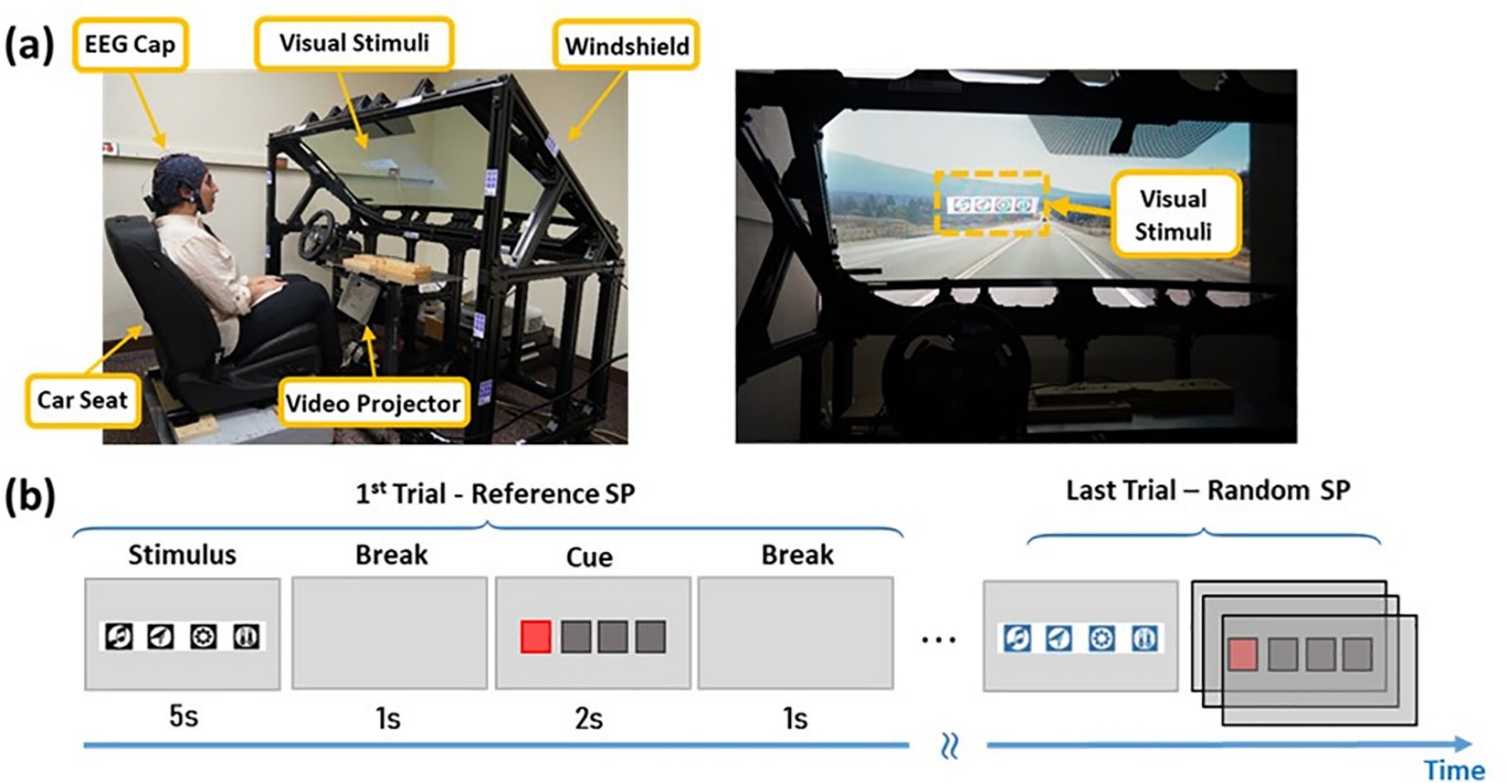
Experimental setup and the HUD (a) and a simplified diagram of experimental procedure (b). The individual pictured in this figure has provided written informed consent (as outlined in the PLOS consent form) to publish their image alongside the manuscript.

This study used a windshield equipped with a diffusion film set at an angle of 35° from the Z-axis, along with a video projector (Epson EB-1795F) featuring a 120 Hz refresh rate to display visual stimuli on the windshield. To eliminate the ghosting effect in the HUD, the angular separation (Δα) between the two images was set to be less than the angular resolution of the human eye, typically in the range of 0.013° – 0.020°. The experimental setting is shown in Fig 4(A). Experiments were conducted in a room without windows to minimize environmental influences on the experimental results.

Luminance measurements of the windshield were obtained using the Photone Grow Light Meter application installed on a smartphone, with the smartphone securely positioned using a holding stand. The application’s accuracy was validated against various lux meters, demonstrating an average error precision of around 3 percent. Despite adjusting the light direction to a critical angle, a substantial portion of the light is transmitted through the glass, resulting in a luminance outside the windshield of 1100 lux, while the light reflected from the surface measured only 80 lux.

### B. Visual stimuli

The stimuli were four targets that flickered at 6.67, 7.5, 8.57, and 10 Hz using a square wave, as shown in Fig 4(B). Each target icon represented a different in-vehicle feature, such as music, navigation, settings, and temperature and was enclosed within a white square box to increase contrast for the participants. We used Python (PsychoPy) to render graphical icons and accurate target frequency [36] on a dedicated PC configured with an Intel Core i7 CPU, 8GB DDR3 RAM, and NVidia GeForce GTX 260 graphic card.

### C. EEG recording

EEG was recorded with a cap during the experiment, while a video projector displayed stimuli on the screen. The 16-channel g.Nautilus (g.tec, medical engineering GmbH, Austria) EEG headset was used in this study to record brain waves. We used electrodes with injected gel at each electrode location. Conductive paste (NuPrep Gel) was applied between the electrodes and the skin to reduce impedance below 5kΩ, minimizing vulnerability to electrical artifacts or movement. The EEG signals were sampled at 500 Hz and transferred to a host computer using Bluetooth connectivity. The electrodes were placed based on the international 10–20 system at Fz, Cz, CPz, P1, Pz, P2, PO3, POz, PO4, PO7, PO8, O1, Oz, O2, TP9, and TP10. The reference electrode was connected to the mastoid, and the ground electrode was connected to AFz.

### D. Experimental protocol and human participants

In this experiment, participants completed four sessions on the same day. They undertook the first session, comprising an investigation with an LCD monitor, and then the remaining three sessions with a configuration with a car seat and windshield in the same room. They were asked to adjust car seat about 95 cm from the windshield and EEG system remained on their heads throughout the experiment. Participants had control over break times between sessions and could pause stimuli anytime during the experiment. Each session was further subdivided into multiple sets. In each set, we changed one parameter at a time to investigate its impact on the SSVEP relative to the reference. We repeated this process for all the parameters under investigation. In each trial, four-icon visual stimuli were prompted five times, resulting in 20 trials for each set. Trials lasted for 9 seconds, during which a red box appeared as the target prompt for 2 seconds, the screen faded off for 1 second before and after the cue to eliminate visual fatigue, and all icons flickered for 5 seconds. While the order of frequency was random, the sequence of stimuli presentation remained consistent for the same subject but was shuffled for other subjects. Participants were instructed to direct their gaze to the target as soon as the target prompt appeared on the screen (Fig 4). Participants were also asked to avoid jerky head and neck movements and minimize eye blinking during the stimulation. Four stimuli flickered simultaneously on the windshield during stimulation intervals and were off during the rest periods, leaving the screen black.

The Institutional Review Board at the University of Connecticut Health Center reviewed and approved the study protocol, and participants provided written informed consent before participating in the study. After reading the participant information sheet and signing a consent form, ten healthy adults (eight males and two females) with self-reported normal or corrected-to-normal vision and no history of central nervous system abnormalities were recruited.

## IV. Signal processing method

### A. Signal pre-processing

All 16 EEG channels were used for analysis. In each trial, we segmented data into idle time and stimuli time. The idle time was taken from 3 seconds before the stimuli onset, and the stimuli time is defined as the stimulation duration in each experiment. We expected the most significant changes in the EEG signal during stimulation to occur at the target stimulation frequency. All segments were filtered with a notch filter to remove powerline interference at 60 Hz; also, a bandpass filter between 4 and 40 Hz was applied to the EEG signals to isolate the frequency range of interest for SSVEP detection [37].

To investigate the impact of SPs on SSVEP, we evaluated SSVEP response using PSDA, CCA, and FBCCA algorithms, which are well-established and commonly used methods among several reported SSVEP detection algorithms and were deemed sufficient for our study’s objectives without requiring more sophisticated methods. Also, we computed signal SNR to assess the signal’s power at the target frequency range for each channel before and after the presentation of stimuli. Detailed explanations of each method are provided in the followings.

**PSDA:** The Welch method was used to calculate the Fourier coefficient with a Hanning window of 1-second length and 50 percent overlap. The averaged power was calculated at all EEG channels in idle time (representing baseline value of spontaneous EEG activity) and stimuli time to find difference increments in SSVEP SNR due to stimuli. The spectral power of the EEG decreases as the frequency increases; therefore, only the sum of the first and second harmonics was considered for the SSVEP SNR. The SSVEP SNR was calculated as the ratio of the power of the signal and the mean value of the six adjacent points, as shown in Eq 2.

$$SSVEP\;SNR=\frac{6\times y(f)}{\sum_{k=1}^{3}y(f+0.25k)+y(f-0.25k)}+\frac{6\times y(2f)}{\sum_{k=1}^{3}y(2f+0.25k)+y(2f-0.25k)}$$ (2)

The SSVEP SNR was averaged over trials within each block to determine stimulus-induced power changes at the selected frequency band.

**CCA:** Unlike machine learning approaches that require training on labeled data, CCA does not need prior training. It directly analyzes the correlation between EEG signals and visual stimuli frequencies, making it efficient for identifying frequency-specific responses without the need for extensive training data. CCA works on two sets of multidimensional variables. Variables in one set are the multi-channel EEG signals, ***x***[*n*], and in the second set are reference templates based on target stimulus frequencies and their harmonics ***y***_***f***_. The fundamental, 2^nd^ and 3^rd^ harmonics of the target frequency were used as simulated reference templates as follows [16,38,39]:

$$y_{f}=\left[\begin{array}{c} sin\;(2\pi ft)\\ cos\;(2\pi ft)\\ \vdots \\ sin\;(2\pi N_{h}ft)\\ cos\;(2\pi N_{h}ft) \end{array}\right],t=\frac{1}{f_{s}},\frac{2}{f_{s}},\cdots ,\frac{N}{f_{s}}$$ (3)

where *N*_*h*_ is the number of harmonics, *N* is the number of sampling points, and *f*_*s*_ is the sampling rate of the EEG signal. CCA tries to find pairs of linear transformations (***w***_*x*_, ***w***_*y*_) for the two sets of variables such that they maximize the canonical correlation, ρ, between multidimensional variables ***x***[*n*] and ***y***_***f***_, where *n* = 0,⋯,*N*−1 is the signal length [15]. Weight vectors of ***w***_*x*_ and ***w***_*y*_ can be found through Eq 4 where they reflect the SSVEP response characteristics under different stimuli configurations.

$$(w_{x},w_{y})=\underset{w_{x},w_{y}}{\mathrm{argmax}}\left|corr(x[n]w_{x},y_{f}w_{y}\right|$$ (4)

Using weight vectors, the correlation coefficient was calculated as in Eq 5 for all target frequencies:

$$\rho ={max}_{w_{x},w_{y}}\frac{w_{x}^{T}E[x[n]y_{f}^{T}]w_{y}}{\sqrt{w_{x}^{T}E[x[n]x^{T}[n]]w_{x}w_{y}^{T}E[y_{f}y_{f}^{T}]w_{x}}}$$ (5)

Subsequently, the target frequency with the highest correlation was regarded as the detected frequency in the SSVEP as follows:

$$detected\;frequency=\underset{i}{\max}\rho_{i},\;i=1,2,\ldots ,k$$ (6)

Where *ρ*_*i*_ are the result of the CCA for the *k* reference signals.

**FBCCA:** To extract different rhythmic components, we decomposed the signal into subbands by a filter bank analysis. To implement the FBCCA method, Chen et al. [9] proposed the use of band-pass filters to extract discriminative fundamental and harmonic frequencies from the original EEG signals with zero-phase Chebyshev Type IIR filters. In their approach, the n^th^ subband started from the frequency at *n*×*k* Hz (where *n* is an index of the subbands and *k* is the starting frequency) and ended at a fixed maximum frequency (40 Hz in the configuration). In the implementation of bandpass filtering, an additional bandwidth of 2 Hz was added to both sides of the passband for each subband.

**ITR:** In addition to classification accuracy, this paper also estimated the ITRs for each frequency. ITR, one of the most commonly used evaluation criteria for the performance of a BMI system, is calculated as Eq 7:

$$ITR=\frac{60}{T}\left[\log_{2}\;N+p\;\log_{2}\;p+(1-p)\log_{2}\left(\frac{1-p}{N-1}\right)\right],$$ (7)

where *N* is the number of targets, *p* is the mean accuracy averaged over all targets, and *T* (seconds/target) is the time for a selection.

### B. Statistical evaluation

We conducted statistical analysis to compare the SSVEP accuracies for different SPs using either a pairwise t-test or a Wilcoxon signed-rank test, depending on whether the data from the two groups were normally distributed. A significant difference between the two groups indicates that the tested SPs have an impact on the SSVEP detection performance relative to the reference SP. Such differences demonstrate the sensitivity of SSVEP to external stimuli or experimental manipulations.

### C. Channel selection

**Fig 5:**
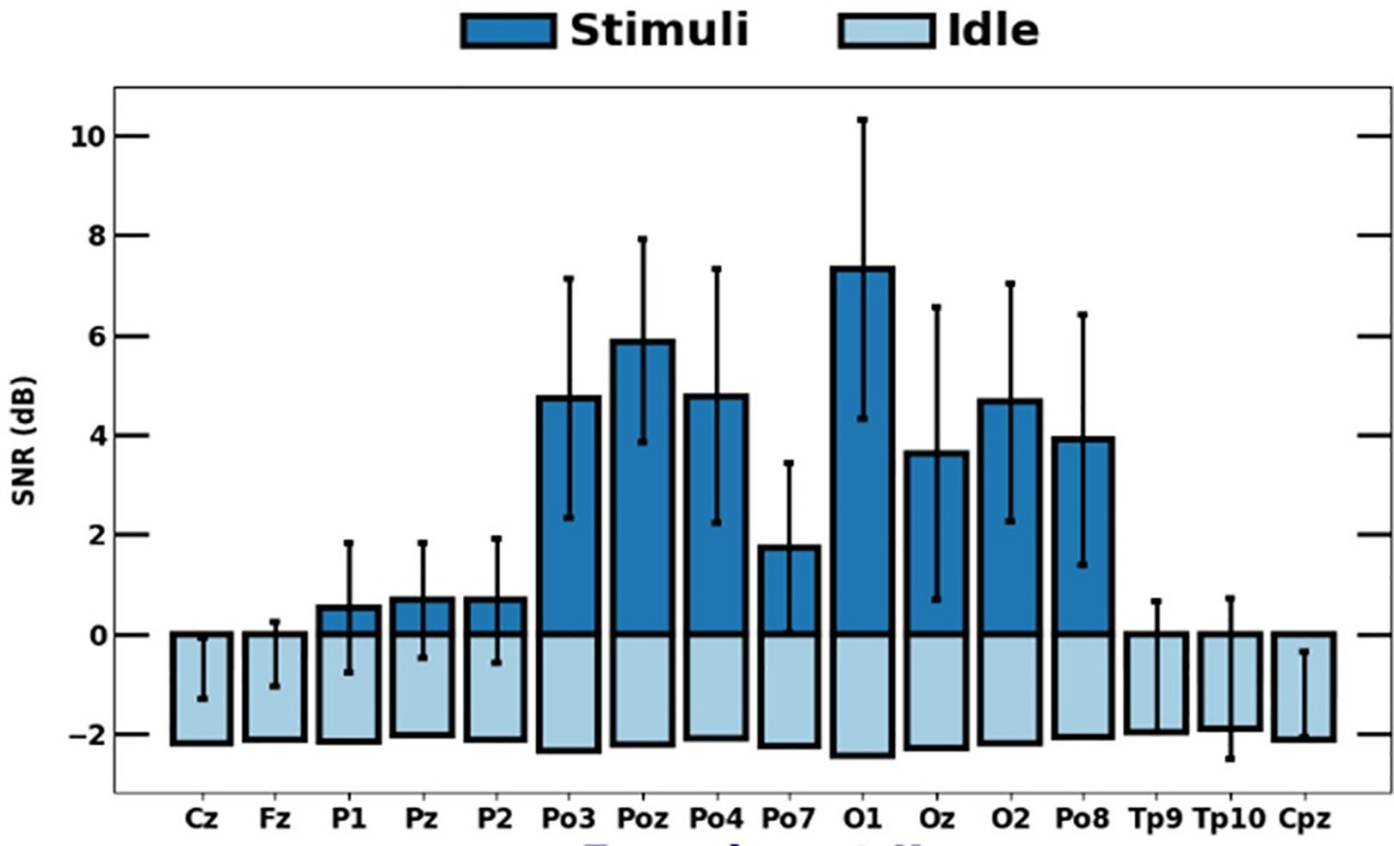
Comparison of channel SNR during idle and stimuli time.

Expanding the number of EEG channels can improve SSVEP detection performance; however, it is essential to identify channels minimally influenced by visual stimuli to avoid confusion during the analysis, particularly for PSDA [40]. Thus, we calculated SNR during idle and stimuli onset states and averaged the results across all trials to find significant changes in each channel. Fig 5 shows the spatial distribution of signal activities over 16 channels. A pairwise t-test on all trials revealed a significant difference (p < 0.01) in all channels between idle and stimuli time. Notably, SNR during stimulation significantly increased in eight channels, PO3, POz, PO4, PO7, O1, Oz, O2, and PO8, compared to the remaining channels. Therefore, we employed these channels for further analysis.

## V. Results

### A. Display device and stimuli SNR

**Fig 6:**
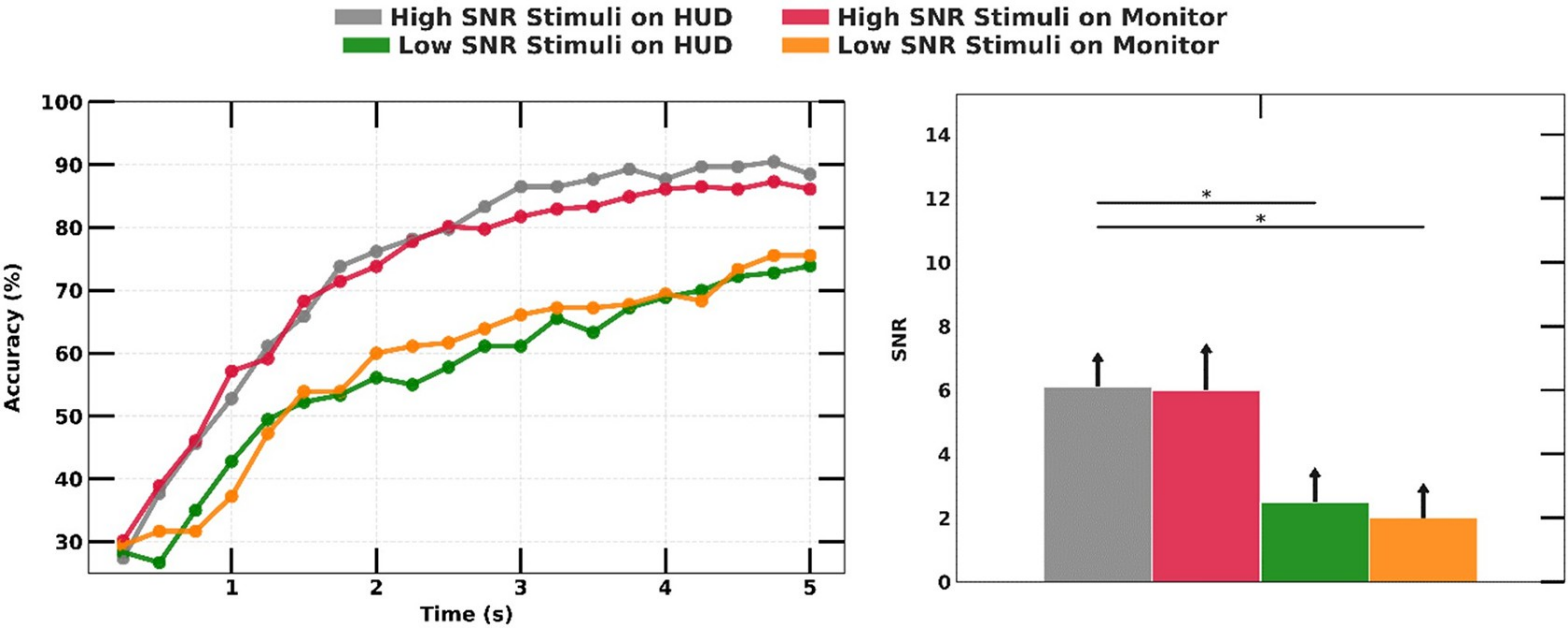
SSVEP detection accuracy over experiment duration with different devices and stimuli SNRs (left) and comparison of measured SSVEP magnitude.

We compared the SSVEP SNR between the reference stimulus displayed on the monitor and the HUD in two conditions of low and high SNR visual stimuli. The luminance of stimuli (both low and high SNR) measured from the eye box was 38 lux for the HUD and 10 lux for the monitor. However, the difference in SSVEP SNR and accuracy between the monitor and the HUD was negligible, with less than 0.5 dB and 4% variation in both low and high SNR stimuli. Across all trials, the high SNR stimuli resulted in an increased SSVEP SNR by 3.6 dB (p-value < 0.01) on the HUD and 4 dB (p-value < 0.01) on the monitor compared to low SNR stimuli (the right graph in Fig 6). Asterisks above the bars in SSVEP SNR plots denote significant differences between tested SPs and the reference on the windshield. We used *P < 0.01 and **P < 0.05 as significance levels to reject the null hypothesis. The same trend observed in SSVEP SNR was also noted in SSVEP accuracy using the CCA algorithm, where stimuli with high SNR stimuli increased accuracy by 13% (the left graph in Fig 6).

### B. Duty cycle

**Fig 7:**
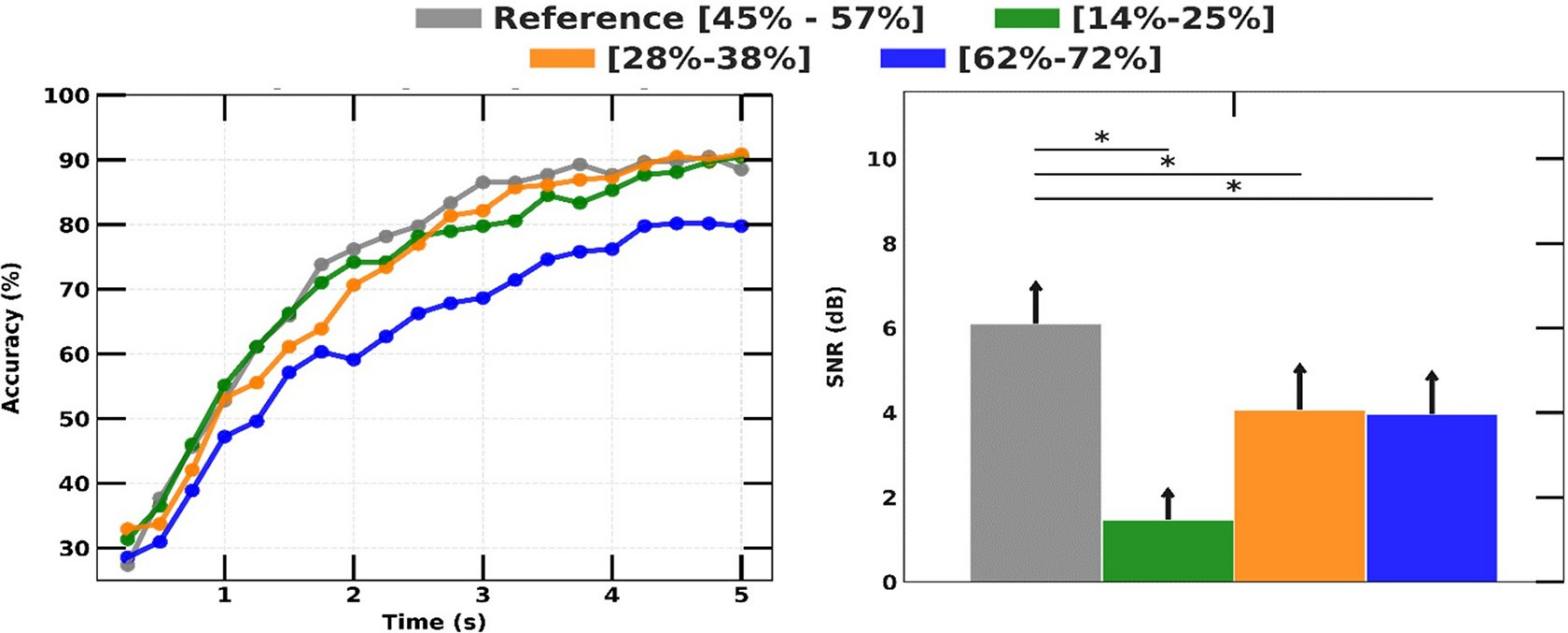
SSVEP detection accuracy over experiment duration with different duty cycles (left) and comparison of measured SSVEP magnitudes (right).

The relationship between the duty cycle of visual stimuli and SSVEP response was investigated by comparing it with the reference stimulus. Based on the SNR of SSVEP magnitude analysis, the duty cycle bins of [62–72%] and [28–38%] exhibited a decrease in SSVEP SNR by about 1.7 dB (p<0.01), while the bin of [14–25%] showed the lowest SSVEP SNR (the right graph in Fig 7). The SSVEP accuracy measured by the CCA across all time intervals for the reference stimulus (bin [45–57%]), consistently showed better results, with a final accuracy of 88.5 ± 4.4%. On the other hand, the SSVEP detection accuracy of the duty cycle bin from [62–72%] for all time intervals was the lowest and achieved an accuracy of 79.8 ± 3.6% with 5 seconds of experiment time. A noteworthy discovery is that, despite exhibiting the lowest SSVEP SNR, the bin of [14–25%] did not exhibit the lowest accuracy when implementing the CCA algorithm. This finding illustrates a complex relation between the duty cycle of stimuli and SSVEP magnitude.

### C. Location and array configuration

**Fig 8:**
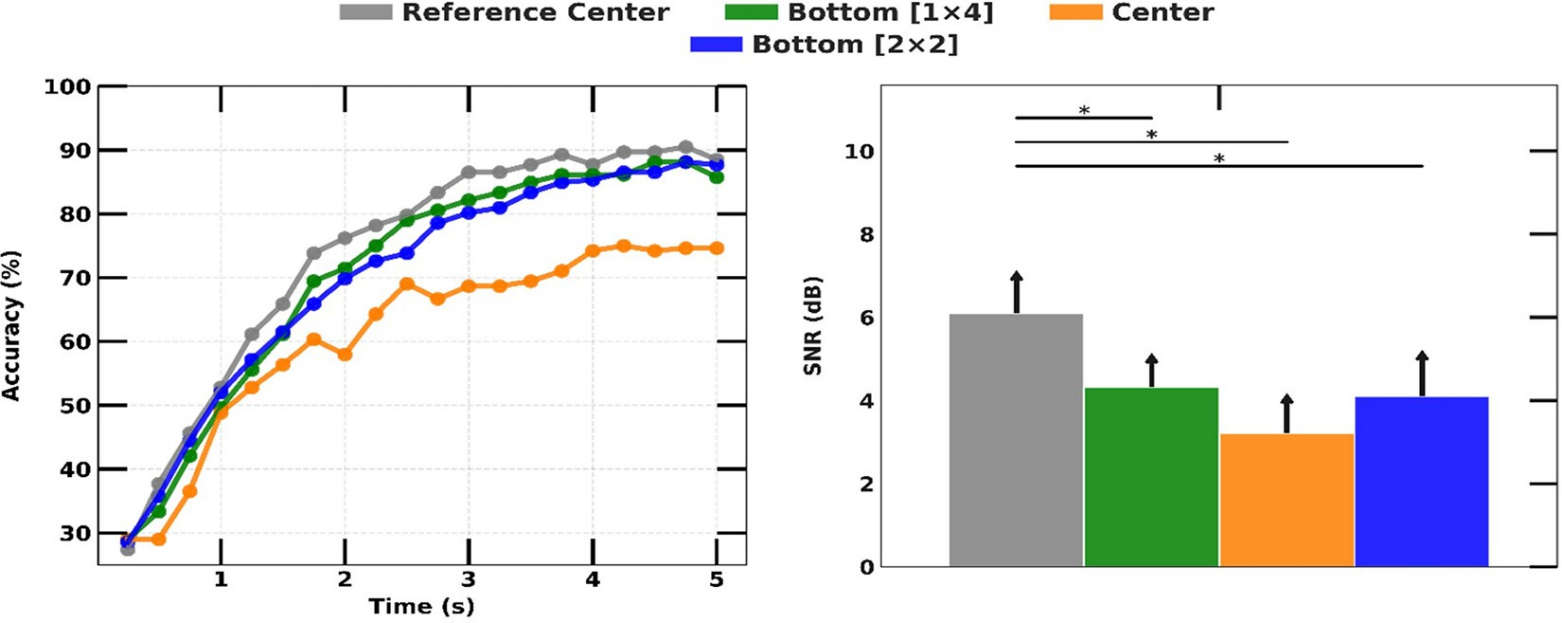
SSVEP detection accuracy over experiment duration with different array configurations and locations (left) and comparison of measured SSVEP magnitudes (right).

The 1×4 array configuration exhibited the highest SNR of SSVEP at the center locations. A decrease of 1.7 dB (p-value < 0.01) in the SNR of SSVEP was observed for the 1x4 array located at the bottom of the screen. The center 2x2 array showed the lowest SNR with a decrement of 2.9 dB (the right graph in Fig 8). This trend was also evident in the SSVEP accuracy, with the central and bottom locations for both array configurations achieving accuracies of approximately 73.9 ± 10.9% and 85.1 ± 9.7%, respectively. The behavior of the bottom location with a 2×2 and 1×4 array was very similar in both CCA and SSVEP SNR analysis.

### D. Icon color

**Fig 9:**
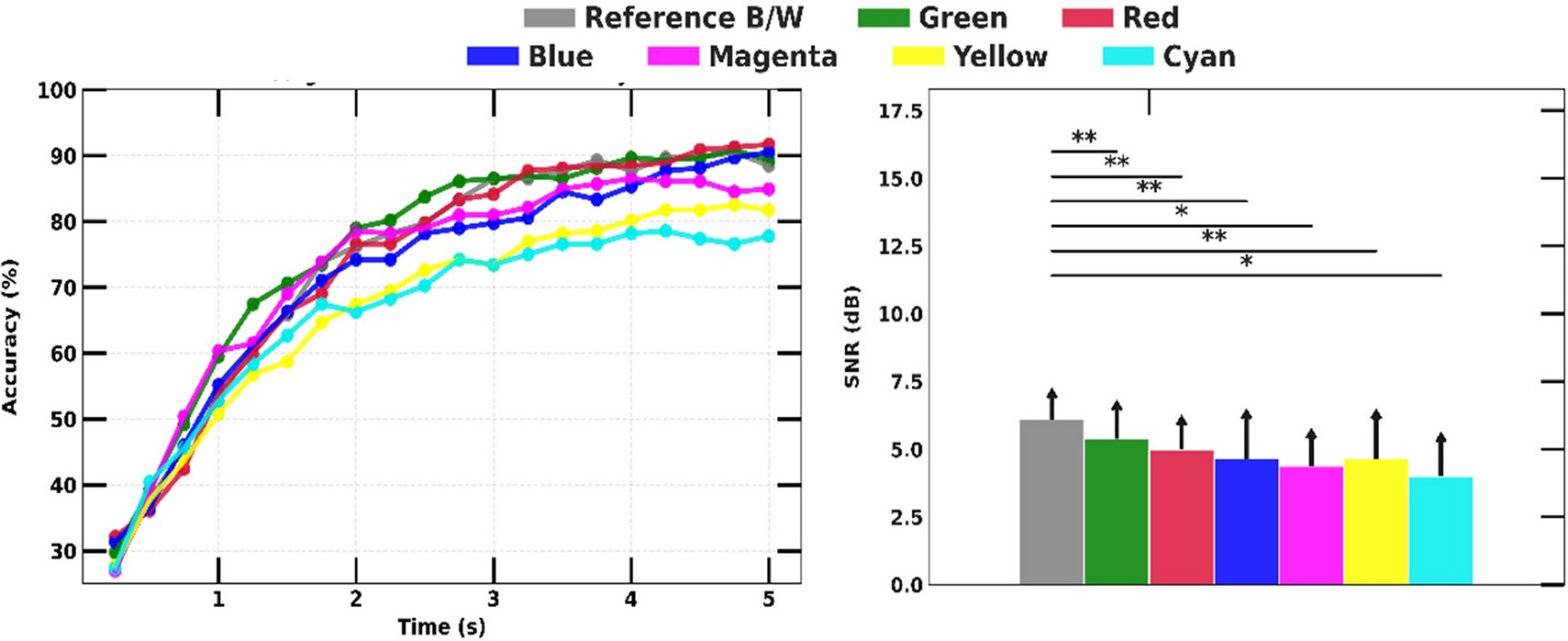
SSVEP detection accuracy over experiment duration with different colors of the icon (left) and comparison of measured SSVEP magnitudes (right).

Our experiment demonstrated the reference stimulus-evoked highest SSVEP SNR (6.1 dB) and followed by the stimuli in green and red colors, with an average of 5.4 dB and 5 dB, respectively (the right graph in Fig 9). However, there was a discrepancy between high SSVEP SNR and high SSVEP detection accuracy. For example, despite having 0.71dB less SSVEP SNR than the reference, the green icon exhibited the highest SSVEP detection accuracy in the first 3 seconds and ultimately achieved similar accuracy to the reference in 5 seconds, with an accuracy of 89% ± 2.1%.

### E. Icon size

**Fig 10:**
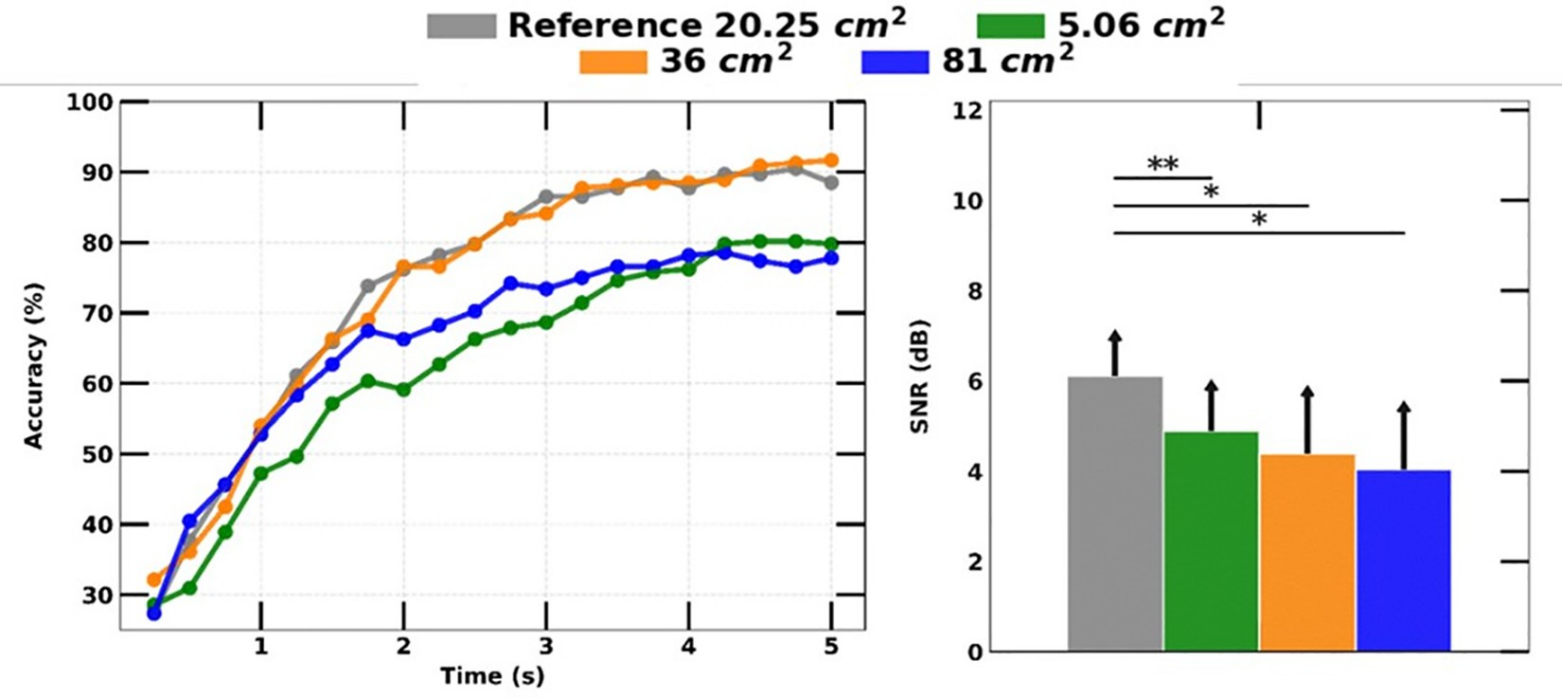
SSVEP detection accuracy over experiment duration with different icon sizes (left) and comparison of measured SSVEP magnitudes (right).

We found a non-linear relationship between icon size and SSVEP detection accuracy. Unexpectedly, as the icon size increased, the SNR of measured SSVEP decreased by 1.2, 1.7, and 2.1 dB (the right graph in Fig 10). The SSVEP detection accuracy showed an increase of 3.2% for 36 cm^2^ icon sizes, but a decrease 8.7% for the icon size of 5.06 cm^2^. However, the icon size of 81 cm^2^ exhibited the lowest performance, with a 10.7% drop in accuracy.

### F. Stimuli frequency

**Fig 11:**
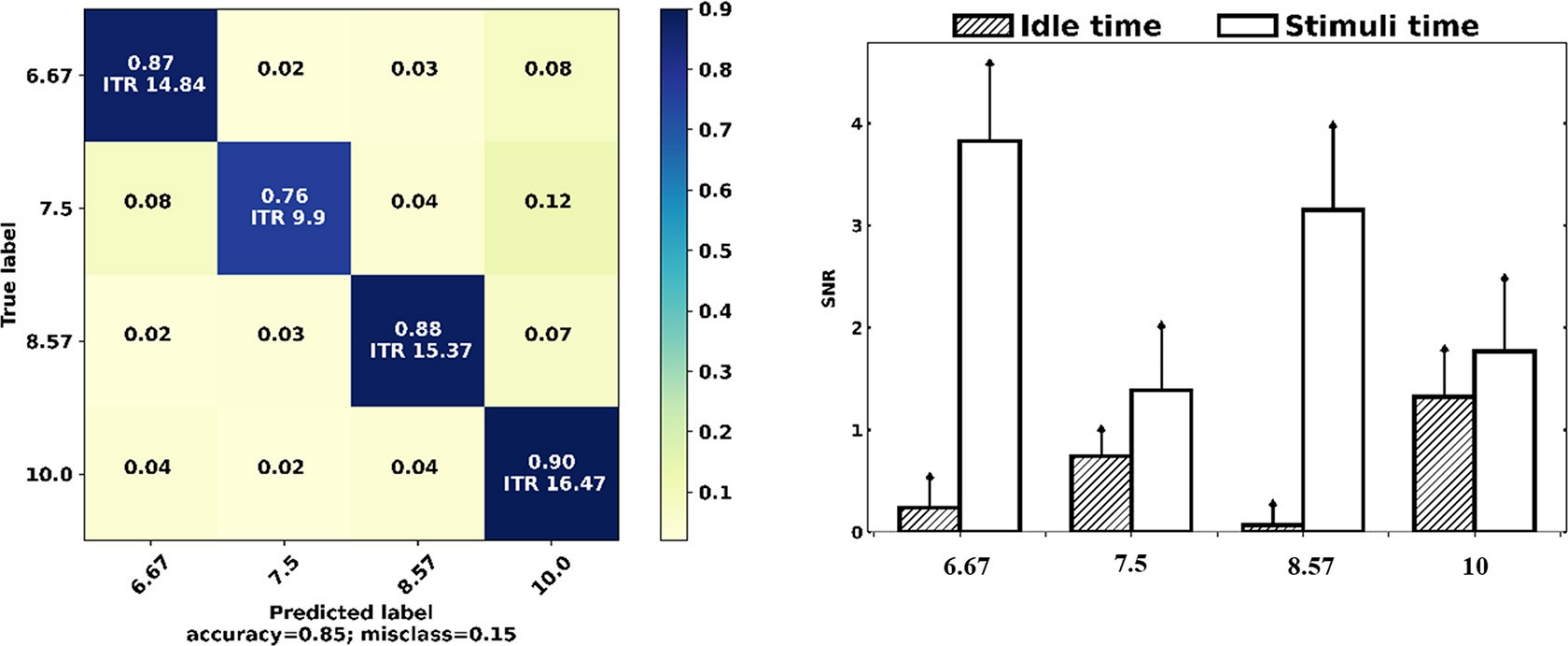
Confusion matrix (left) and SNR changes (right) based on frequency.

As shown in Fig 11, 8.57 Hz stimuli strongly increased SSVEP SNR with 9 dB during stimuli onset, which also presented a high CCA accuracy of 88%, whereas 7.5 Hz stimuli exhibited the lowest SSVEP SNR increment of 4.34 dB and resulted in the lowest ITR of 9.9 bits/min. Further, 8.57 Hz and 10 Hz, with the highest SNR increment, produced the highest ITR.

### G. SSVEP detection accuracy comparison of CCA and FBCCA

We also evaluated the significance of algorithm choice in enhancing the proposed system’s capability for real-world situations. Specifically, FBCCA was intended to investigate its potential to accelerate detection compared to the CCA method. In order to provide a comprehensive comparison, Table 5 summarizes the accuracy of each SP for 1.5 and 3 seconds of data. Although FBCCA managed to improve the overall accuracy, it’s important to note that the fundamental patterns of the signals remained consistent.

**Table 5:** Acurracy comparison of CCA and FBCCA algorithms.

Type of SPs	CCA Accuracy (%)		FBCCA Accuracy (%)	
	1.5 sec	3 sec	1.5 sec	3 sec
Duty Cycle [14–25]	**66.27**	79.76	**71.39**	82.47
Duty Cycle [28–38]	61.11	82.15	65.14	84.54
*Duty Cycle [45–57]	65.88	**86.51**	71.10	**90.74**
Duty Cycle [62–72]	57.14	68.65	61.18	71.19
Bottom 2×2	61.51	80.15	66.57	81.21
Bottom 1×4	61.11	82.15	66.28	86.58
*Center 1×4	**65.88**	**86.51**	**71.10**	**90.74**
Center 2×2	56.35	68.65	61.72	70.14
Blue	66.27	79.76	70.83	82.96
Red	66.27	84.12	71.20	85.81
Green	**70.63**	**86.51**	**75.43**	**89.96**
Magenta	69.05	80.95	73.94	84.16
Cyan	58.73	73.41	62.83	76.46
Yellow	62.70	73.41	66.71	77.62
Size 5.06 cm^2^	57.14	68.65	61.43	70.81
*Size 20.25 cm^2^	65.88	**86.51**	**71.10**	**90.74**
Size 36 cm^2^	**66.27**	84.12	70.68	87.95
Size 81 cm^2^	62.70	73.41	67.61	75.45

* Reference Icon.

## VI. Discussion

In this study, we investigated the effects of various SPs on SSVEP response, including HUD, frequency, duty cycle, location, array configuration, icon color, icon size, background images, and SNR of stimuli. Many SSVEP studies have used LCD monitors to establish baseline performance metrics. This study benchmarks HUDs against these metrics to identify their unique challenges and advantages, to aid optimizing HUD designs for in-vehicle applications. This is the first comprehensive study to investigate the impact of systematic changes in SPs, especially in the context of using a HUD, on the SSVEP response in a vehicle environment.

### A. Visual stimuli frequency

**Fig 12:**
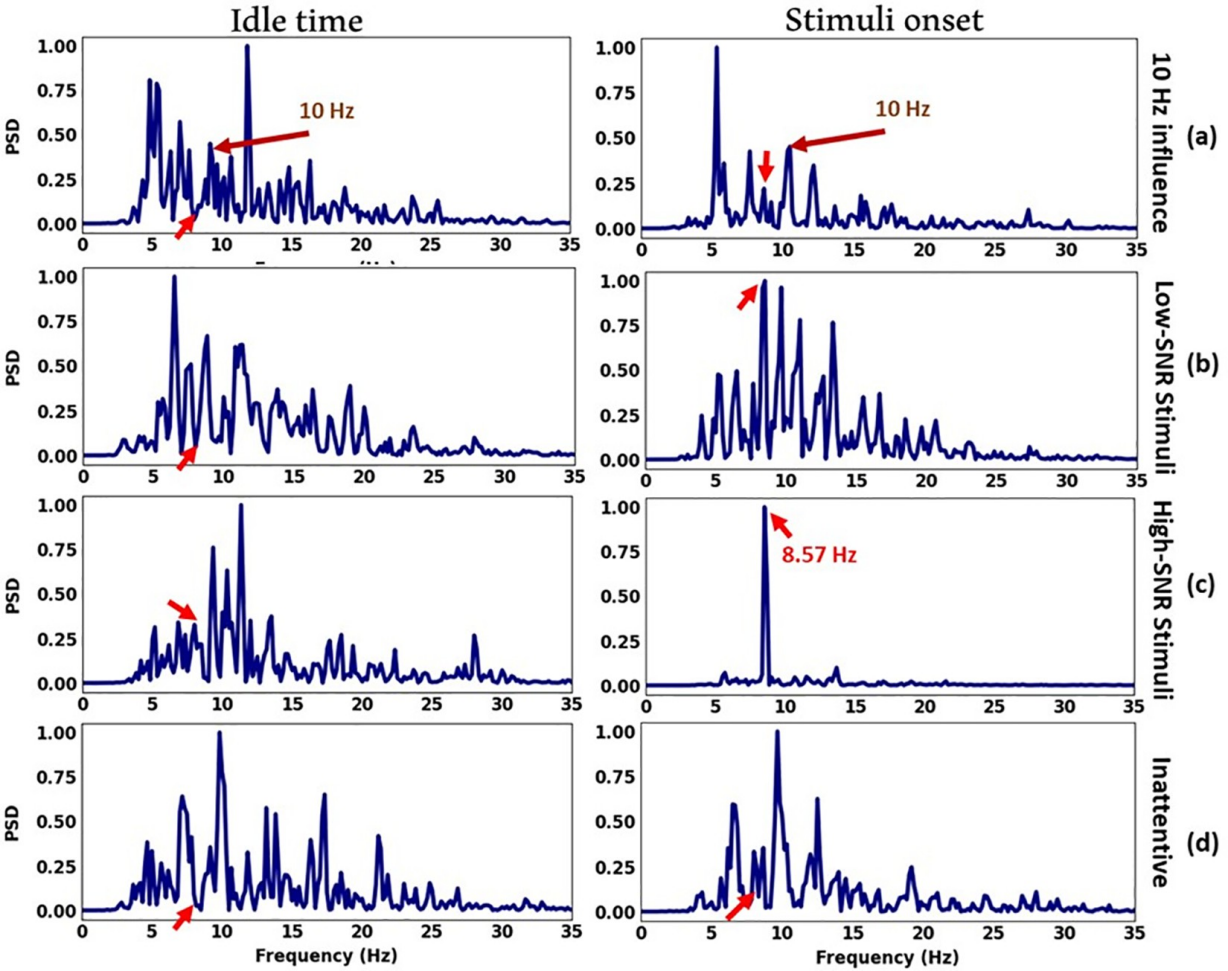
Average over five trials for one participant and for channel Oz to demonstrate the impact of (a) 10 Hz unwanted frequency, (b) low SNR stimuli, and (c) high SNR stimuli in the presence of 8.57 Hz stimuli on the SSVEP signal, (d) a single inattentive trial by the same participant. The EEG signals recorded before stimuli onset are presented on the left column, while those recorded after stimuli onset are shown on the right column. Red arrows point to stimuli frequency at 8.57 Hz.

We used a frame-based stimulus design based on the monitor refresh rate to ensure frequency stability. The selected frequencies were from the low-frequency band (<12 Hz), where the SSVEP response is significantly stronger to detect [41]. In our experiments, 10 Hz stimuli evoked the strongest SSVEP response, which led to the highest detection accuracy using the CCA algorithm. This confirms that the visual cortex responds to flickering stimuli at resonance frequencies (i.e., 10-, 20-, 40-, and 80-Hz) more strongly than other frequencies [42]. As depicted in Fig 12A, for some participants, a persistent unwanted frequency at 10 Hz was observed before and after stimuli onset, even in the absence of 10 Hz stimuli. This phenomenon suggested that SSVEP is susceptible not only to its resonance frequency but also to integer multiples and sub-harmonic resonance. Multiple objects in a cluttered visual scene compete for neuronal representation while driving, and for instance, imperceptible oscillations of 80 Hz in the ambient could evoke identical harmonic and subharmonic stimulus frequencies of 10 Hz in the SSVEP [43]. Lower SNRs of stimuli can exacerbate this issue by allowing other frequencies in the ambient environment to modulate with the target frequencies, which can confound the classifier. For instance, in the presence of 8.57 Hz stimuli with low SNR, as shown in Fig 12B, the signal could barely compete with the 10 Hz unwanted noise. In contrast, Fig 12C demonstrates that high SNR stimuli can effectively produce high-quality SSVEP signals. It is also important to consider the impact of inattentive SSVEP signals on the classification accuracy. Inattentive SSVEP signals can occur when the participant is not fully engaged with the visual stimulus, resulting in weaker SSVEP responses (Fig 12d). These weaker responses can lead to lower SSVEP SNRs, making it difficult to distinguish between target and non-target stimuli.

### B. Duty cycle

Our study found that adjusting the duty cycle, which changes the proportion of ON and OFF states in visual flicker, significantly impacted SSVEP response due to changes in the stimuli’s energy distribution. Specifically, we observed that higher duty cycles (i.e., bin [62–72%]) led to a decline in SSVEP strength. This is because as the duty cycle increased to higher values, the DC component acted as a constant luminance source, strongly inhibiting polarity change in the two sides of the eye membrane [44]. As shown in Fig 7, the duty cycle bin [62–72%] resulted in significantly lower SSVEP detection accuracy compared to other duty cycle bins.

A prior study has shown that uncomfortable flickers (low duty cycle, prolonged OFF state) could draw more attention than higher duty cycles [45]. Thus, in the ideal setting, lower duty cycles may elicit higher SSVEP detection accuracy. Our results also confirm this, as the duty cycle bin [14–25%] resulted in weaker SSVEP SNR, but SSVEP accuracy was comparable with the reference duty cycle bin [45–57%], which consistently elicited strong SSVEP responses throughout the experiment. These findings suggest that the duty cycle of visual flicker is an important factor to consider when designing SSVEP-based BMI systems and that a balanced duty cycle that maximizes SSVEP response while minimizing discomfort is critical for optimal performance.

The concept of on/off states, as seen in duty cycles, can be extended to recent developments utilizing aperiodic flickering visual stimuli in the form of code-modulated visual evoked potentials (c-VEP) [46,47]. Unlike SSVEP, which relies on periodic flickering stimuli, c-VEP employs aperiodic, binary (on/off) visual stimulation patterns or codes. These visual stimuli are modulated by predetermined pseudorandom code sequences, such as maximum length sequences (m-sequences). Each stimulus is linked to a unique code, eliciting a distinct brain response pattern. This aperiodic nature offers greater flexibility in optimizing the temporal properties and duty cycles of stimuli in c-VEP systems. A significant advantage of the c-VEP approach is that the training of the model is independent of the number and complexity of targets, reducing calibration time [48].

### C. Location, array configuration, and FOV

The performance of SSVEP is influenced by the angular degree of a light ray entering the eye pupil. By directing attention overtly to different target positions on the screen, the stimulus was reflected at different locations on the retina [35]. Human observers are considerably more sensitive to light entering through the center of the pupil than to off-center rays. The evoked potential response is reportedly greatest at the fovea, decreasing as a Gaussian function of width to about 5° centered at the point of fixation [49]. Our experiments showed that the center and bottom locations, which lie within 5° of the fovea, showed the highest SSVEP detection accuracy while being natural to the driver’s eye and not causing distraction.

We found that the arrangement of the icons on the windshield significantly affected SSVEP response. To minimize mutual interference among the stimuli, we arranged them at an appropriate distance, where only one stimulus appeared in the FOV. Studies have shown a significant decrease in amplitude when the competing stimulus is closer than about 4.5° of visual angle [49,50]. Although this view angle was sufficient for the 1×4 array (around 6.0°), it was not enough for the 2×2 array configuration (around 2.0°), and the SNR and accuracy were suppressed significantly. Gao et al. [51] recommended maintaining a visual angle from 8° to 12° for better performance. These findings highlight the importance of proper stimulus arrangement and positioning in SSVEP-based BMI systems for optimal performance.

### D. Icon color

Colors can have different effects on the human eye, impacting the SSVEP response. Firstly, the contrast of the visual stimulation can impact neuron firing rates, with stronger neuronal activity as the contrast intensifies [5]. Our results confirm that colors with higher contrast ratio (B/W (21:1), Green (5.13:1), and Red (3.99:1)) elicit stronger SSVEP response, as indicated by higher SSVEP SNR and SSVEP detection accuracy, compared to lower-contrast colors such as Cyan (1.45:1) and Yellow (1.07:1). However, it is important to note that the use of Red may not be suitable for all individuals due to its reported potential to cause visual fatigue and epilepsy [17]. Secondly, the human eye perceives colored light to be brighter than white light of the same luminance due to the HK effect [11]. Therefore, colors such as magenta (3.13:1), which appears brighter than blue (8.59:1) in the same brightness [18], can induce a stronger SSVEP response. Lastly, brightness illusions can modulate the pupillary light response, with some colors performing better despite being equiluminant [11]. For example, the color yellow performed better than cyan despite having a lower contrast ratio and three times less HK magnitude [18].

These findings suggest that the careful selection of colors is important in optimizing SSVEP performance, taking into consideration factors such as contrast ratio, brightness, and potential discomfort or adverse effects. The use of higher-contrast colors, such as B/W and Green, may result in stronger SSVEP responses and is advantageous in SSVEP-based BMI systems.

### E. Icon size

The size of the stimulus is known to have a direct relationship with the amount of light that enters the eye box, which in turn increases the amount of light information detected in the visual sensory circuit. Increased light information results in an escalation of neuronal activity and can cause an increase in amplitude-evoked responses. Our findings are consistent with previous evidence [12] and indicate that, in the majority of trials, SSVEP amplitude increased with larger stimulus size. Additionally, we observed that SSVEP detection accuracy increased with bigger stimulus sizes of 36 cm^2^ and decreased with smaller stimulus sizes of 5.06 cm^2^. These results suggest that stimulus size is an important factor to consider when designing SSVEP-based BMI systems and that the optimal size is likely to be a balance between maximizing the amount of light entering the eye and minimizing the discomfort caused by excessively large stimuli. Further research is needed to determine the ideal stimulus size for different applications and populations. Overall, this study contributes to the development of SSVEP-based BMIs for vehicle applications and provides insights into the optimal configuration of SPs for achieving high accuracy.

### F. Study limitations

The findings of the study suggest that SSVEP-based BMI that uses a HUD has great potential to replace distractive screens and buttons for in-vehicle feature controls. However, there are still some limitations that need to be addressed. For instance, the study was conducted with only ten participants, and further studies with a larger sample size are needed to verify the results of this study. In addition, this study did not investigate factors in a natural environment, such as different driving and road conditions, which may affect the accuracy of the SSVEP-based BMI system. Also, it is important to assess the impact of driving-related motion artifacts in real situation.

Future research should also investigate other important factors that may affect the usability of the system, such as user fatigue, visual distractions, the number of target icons, and the potential for motion sickness. Driving requires divided visual attention to track multiple objects simultaneously that studies should explore how divided attention during driving could affect SSVEP amplitude.

The frequency generation on the monitor wasn’t consistently precise across all frequencies. This might be due to non-linear behavior or imperfections in the monitors, leading to additional unwanted frequencies around the target frequencies. In Fig 3, sharp peaks are seen at 6.67 Hz and 10 Hz, but there are also unwanted peaks at 7.5 Hz. For future studies, we recommend using high-end gaming monitors with consistent specifications to minimize the error and improve the precision of generating the desired frequencies on the monitor.

Our study also highlighted the need for more advanced SSVEP detection algorithms that can overcome the challenges posed by real-world driving scenarios. While the CCA, PSDA, and FBCCA algorithms used in this study are well-established and widely used, they may not be sufficient to achieve high accuracy and reliability in a driving environment where an asynchronous SSVEP approach may be required to discriminate between idle and control states. Therefore, advancements in classification methods and the selection of optimal SPs, incorporating machine learning techniques outlined in previous studies [52,53], could result in increased accuracies and significant improvement of BMI system performance through faster detection of SSVEPs. However, we anticipate that our findings regarding the relationship between SPs and SSVEP detection will largely remain unaltered, ensuring the continued relevance of this research.

Furthermore, this study was conducted in a controlled environment, which helped minimize the impact of external variables and provided a more explicit assessment of the stimulus properties. However, this system is susceptible to external factors such as ambient light, visual distractions, and other environmental conditions in real-world scenarios. These factors must be thoroughly considered and addressed to ensure the system’s effectiveness and reliability.

## VII. Conclusion

This study investigated the effect of SPs on SSVEP-based BMI using a HUD in a simulated vehicle environment. To our best knowledge, this article is the first kind of report that assessed the effect of SPs on transparent glass using windshield HUD and comprehensive SPs that could affect SSVEP response in a vehicle application. In this study, four-icon stimuli controlling in-vehicle features flickered within a typical driver’s field of view while focused on the road. To evaluate system efficiency and find the optimal SP, we investigated SSVEP performance in multiple scenarios and changed properties such as duty cycle, size, color, frequency, SNR, location, background activity, and array configuration. The study results demonstrated that the optimal SP was green color, a duty cycle of bin [45–57%], central location, and a size of 36 cm^2^ (3.6°, 206’) icons. The study also showed no significant difference between the HUD-based and the state-of-the-art LCD-based methods, setting the stage for further exploration and optimization of HUD-based SSVEP vehicle systems that potentially offer reduced driver distraction.

It is worthwhile noting that our experimental results suggest that the SSVEP-based BMI system can achieve an accuracy of higher than 90% in five seconds with the eight channels located in the occipital region with the optimal combination of SPs. Thus, the SSVEP-based BMI system showed great potential to substitute distractive screens and buttons for in-vehicle feature controls.
